# *Cannabis sativa* as an Herbal Ingredient: Problems and Prospects

**DOI:** 10.3390/molecules29153605

**Published:** 2024-07-30

**Authors:** Ayodeji O. Oriola, Pallab Kar, Adebola O. Oyedeji

**Affiliations:** 1 Department of Chemical and Physical Sciences, Faculty of Natural Sciences, Walter Sisulu University, Nelson Mandela Drive, P/Bag X1, Mthatha 5117, South Africa; aoriola@wsu.ac.za; 2 African Medicinal Flora and Fauna Research Niche, Walter Sisulu University, Mthatha 5117, South Africa

**Keywords:** *Cannabis sativa*, cannabinoids, biological activities, cannabis legality, cannabis economy

## Abstract

*Cannabis sativa*, otherwise known as hemp, is discussed to highlight the various problems and prospects associated with its use as an herbal ingredient. The chemical composition of hemp, with classification based on cannabinoid contents, its biological activities, current global scenarios and legality issues, economic importance, and future prospects, are discussed.

## 1. Introduction

**Figure 1:**
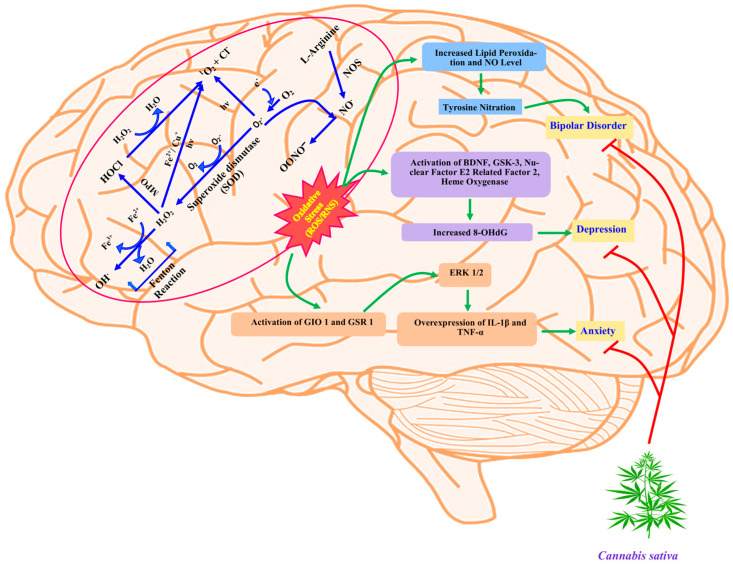
Schematic representation illustrating how the production of free radicals (ROS/RNS) affects biological systems by causing cellular stress and even resulting in various mental illnesses, as well as how *Cannabis sativa* may help to treat these. [HOCl: Hypochlorous acid; H_2_O_2_: Hydrogen peroxide; ^1^O_2_: Singlet oxygen; O_2_^.−^: Superoxide; OH^•^: Hydroxyl radical; OONO^−^: Peroxynitrite; NO^.^: Nitric oxide; Fe^2+^: Iron ion; Cu^2+^: Copper ion; Cl^−^: Chlorine ion; ROS: Reactive oxygen species; RNS: Reactive nitrogen species; NOS: Nitric oxide synthase; MPO: Myeloperoxidase; BDNF: Brain-derived neurotrophic factor; GSK-3: Glycogen synthase kinase 3; 8-OHdG: 8-hydroxy2-deoxyguanosine; ERK: Extracellular signal-regulated kinases; IL-1β, interleukin 1 beta; TNF α: Tumor necrosis factor α].

For the past 5000 years, hemp (*Cannabis sativa* L.), a flowering annual herbaceous crop plant, has been utilized in agriculture. Native to Asia, it is now widely distributed throughout North America, Europe, and Africa [1]. Three species of cannabis were originally recognized: *Cannabis sativa*, *Cannabis indica*, and *Cannabis ruderalis*. However, these species are now recognized as variations of *Cannabis sativa* (var. *sativa*, var. *indica*, and var. *ruderalis*). Currently, *C. sativa* strains are divided into three main categories: industrial hemp, CBD-type hemp, and medical cannabis (high THC) [2,3]. Globally, 147 million people have been reported to consume cannabis. This high level of cannabis consumption is partly attributed to its intoxicative effect [4]. Many people who are only aware of cannabis as a recreational narcotic fail to recognize its huge social, industrial, and economic significance. Hemp, or cannabis, is a versatile crop; in the past, people have planted hemp for its seeds, fibers, essential oils, and medical properties [1]. Despite being a diverse plant with both medical and industrial uses, *C. sativa* is a controversial plant. It is popularly grown worldwide for illicit drug usage (marijuana). During the past few decades, there have been deliberate measures (outlawing) put in place to curb the widespread recreational use of cannabis because of its psychoactive Tetrahydrocannabinol (THC) content. This has significantly and unjustly hurt the hemp industry. Worldwide, hemp seeds and inflorescence are widely used as dietary sources and ingredients in food supplements. Rich in physiologically active, nonpsychoactive cannabinoids, hemp inflorescence contains cannabidiol (CBD), cannabinol (CBN), and cannabigerol (CBG), which have strong activity against reactive oxygen species (ROS) as antioxidants (Figure 1); thus, they may exhibit anticonvulsant, anxiolytic, and spasmolytic properties, among others [5]. With its deliciously nutty flavor, hempseed is an excellent source of fiber, minerals, vitamins, and vital fatty acids. It also contains important amino acids, which are found in the highly digestible protein albumin and edestin [6]. Most hempseed oils (>90%) contain polyunsaturated fatty acids [7], which are well known for their protective properties against inflammatory disorders, cancer, and cardiovascular disease [8,9]. Since 2017, hemp has also been used to make beer. This resulted from the federal ban on marijuana and the relaxation of regulatory barriers pertaining to the use of cannabis plant portions other than the seed, which contains the psychoactive Δ^9^-Tetrahydrocannabinol (Δ^9^-THC) [10]. This review provides a comprehensive analysis of *Cannabis sativa* as a useful herbal ingredient in food and medicine, and its biological properties, legality issues, and economic prospects.

## 2. Methodology

The study involved an open (no date restriction) literature review of work published on *C. sativa* concerning its use as an herbal ingredient, using various scientific search engines such as Google Scholar, Mendeley Scopus, PubMed, and Science Direct. The following inclusive criteria were used in the study: *Cannabis sativa*, hemp, or Cannabis (its active ingredients and cannabinoid contents), and medicinal values, non-medicinal values, biological activities, and economic prospects.

## 3. Industrial Uses and Processing of *Cannabis sativa* or Hemp

Hemp industries use known hemp cultivars and other plant components to obtain the necessary components and the desired end products [11]. Three distinct parts of hemp are used: fibers, seeds, and flowers [3]. Industrial hemp uses seeds and fibers, while CBD-type hemp uses flowers only [12].

### 3.1. Hemp Fiber

Approximately half of the world’s fiber hemp supply is produced in China, where the industrial hemp fiber market is dominant [3]. *Cannabis sativa* is heavily fertilized to prevent blooming and promote taller stalks for hemp fibers. It is then harvested similarly to hay [3,11]. The optimum density for this form of hemp is 32–37 plants/m^2^ [13]. Plants are kept in storage until the stalks dry out, at which point the stalks are processed by dividing the tough, woody inside of the hurd from the outer fibrous exterior of the bast fibers [3,11]. While the hard fibers can be processed into building materials like particle board and hempcrete, the bast fibers can be used to make products like textiles, rope, or paper [3].

### 3.2. Hemp Seed

The plant is grown similarly to that of fiber for hemp seeds. In a dense seeding arrangement, male and female plants are deliberately sown close to one another [3]. For optimal pollination potential, male plants can be surrounded by female plants. To prevent seed dispersal, harvesting must be conducted rapidly [3]. Hemp oil can be extracted by pressing the dehulled seeds [3]. Thereafter, hemp seeds can be prepared for sale as protein powder, flour, hemp oil, roasted snacks, and/or animal feed [14,15].

### 3.3. Hemp Flowers or CBD-Type Hemp

Hemp with CBD is produced and harvested distinctly. The chemical makeup of hemp changes from tissue to tissue throughout the plant [11]. The female flowers contain significant quantities of CBD and terpenes, which are the desirable ingredients for hemp with CBD [11,16,17,18]. To optimize flower production, plants are widely separated; their heights range from 1.2 to 2.4 m [3]. A distance of 0.9–1.2 m is suitable spacing between hemp plants that are CBD-type [3]. The necessary components must subsequently be extracted from the adult blossoms by thoroughly drying them [3,11]. The cannabinoids are decarboxylated when the flowers are dried [11]. A variety of techniques, such as alcohol extraction, supercritical CO_2_, or liquid hydrocarbons, can be used to extract their compounds [3]. The steps required for alcohol extraction include chilling the ethanol, pumping it into a container containing *C. sativa* plant material, and letting it soak. After the alcohol has been removed from the extract through distillation, a mixture of cannabinoids is left behind that can be used in various applications or suspended in oils. Carbon dioxide is pressurized and placed in a chamber along with the plant material using supercritical CO_2_. By removing the CO_2_, the distillate can be identified, yielding the purest chemical profile. The last method for using liquefied hydrocarbons is to mix plant material with compressed low molecular weight hydrocarbons, and then the hydrocarbons are evaporated. The liquefied hydrocarbon process is well known for requiring appropriate safety measures because the hydrocarbons involved are flammable. Product makers in the region favor supercritical CO_2_ and alcohol extractions out of these three techniques [19,20].

## 4. Hemp Chemical Composition

**Figure 2:**
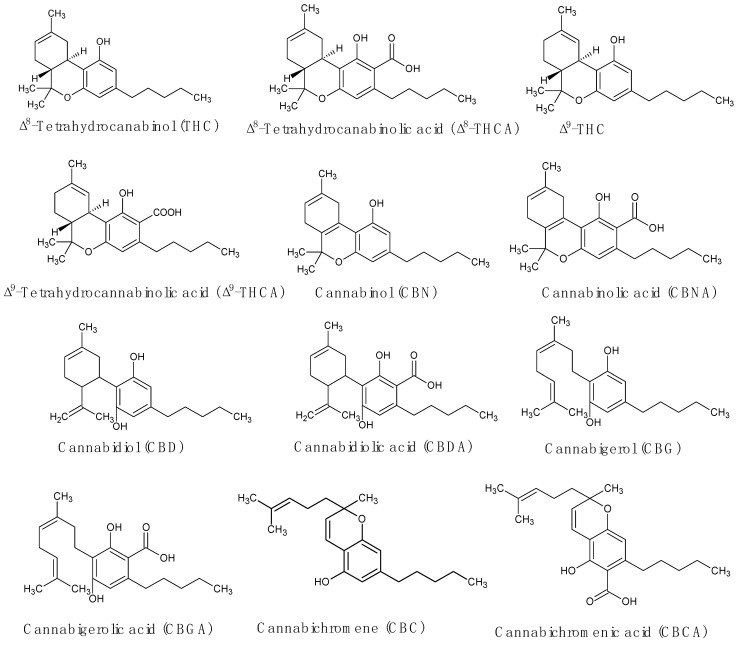
Some cannabinoids from *Cannabis sativa*.

Hemp has about 600 distinct chemical components [11]. Among these, almost 180 are cannabinoids, making up a significant portion of the identified metabolites [21]. According to Głodowska and Łyszcz [21], several of these compounds exist in decarboxylated and acidic forms. THC, CBD, CBN, CBG, and cannabichromene (CBC), as illustrated in Figure 2, are the primary cannabinoids found in hemp [21]. THC and CBD are the two most well-known cannabinoids.

### 4.1. Tetrahydrocannabinol

Tetrahydrocannabinol, also known as Δ^9^-THC, is thought to be the most studied cannabinoid [21]. *Cannabis sativa* has long been prohibited because of its psychotropic qualities. In contrast to CBD-type strains that have less than 0.3% THC, medical cannabis (high THC) strains typically have around 10% THC [21]. The acidic form of THC that is biosynthesized and more frequently found in living plants is called tetrahydrocannabinolic acid (THCA). Due to the double bond between the ninth and tenth carbon, it is known as Δ^9^-THC [22]. Many studies have been conducted to determine the medicinal benefits of Δ^9^-THC, which has been shown to have potential as an appetite stimulant, painkiller, and antiemetic [23]. Mueller et al. [24] claim that Δ^9^-THC is detrimental to cognitive function, particularly in young individuals. It has been demonstrated that Δ^9^-THC influences memory, cognitive speed, and learning [24]. Memory impairment, paranoia, disorientation, and dizziness are among the negative effects that Δ^9^-THC is more likely to cause than CBD. However, when Δ^9^-THC and CBD are mixed, CBD usually counteracts the cognitive symptoms of pure Δ^9^-THC [25].

### 4.2. Cannabidiol

Cannabidiol (CBD) is one of the most significant cannabis molecules in medicine. Anxiety, depression, multiple sclerosis, post-traumatic stress disorder (PTSD), schizophrenia, and dementia are among the psychiatric and neurologic conditions for which CBD has been demonstrated to show a considerable level of effectiveness [18,21,25,26,27]. It has been reported to exhibit antimicrobial, anti-inflammatory, and antioxidant activities [18,26,27]. Certain malignancies, diabetes, and seizures in epilepsy are known to be manageable with CBD [18,25,26]. It has been demonstrated to promote collagen crosslinking, which improves bone fracture healing [28]. According to Whyte et al. [29], treating mice with pure CBD decreased bone resorption. Epidolex, a pure form of CBD, is an FDA-approved pharmaceutical for the treatment of seizures in Lennox–Gastaut syndrome and Dravet syndrome [30].

### 4.3. Other Cannabinoids

*Cannabis sativa* contains other cannabinoids that may have medicinal and other applications. Cannabigerol (CBG), cannabigerolic acid (CBGA), and cannabidiolic acid (CBDA) are chemical precursors of CBD that are included in goods’ total CBD content and are known to have marked biological effects such as anti-inflammatory and antibacterial activities [18]. Young cannabis plants are the ones that typically contain CBC, while the older ones contain CBN [26]. According to Andre [26], the former is known for its sedative, anti-inflammatory, antibacterial, and antifungal properties, while the latter is a sedative byproduct of THC breakdown [26].

### 4.4. Terpenes

Terpenes may be metabolized either primarily or secondarily. According to Iseppi et al. [31], plants require primary metabolites to survive, but they also require secondary metabolites to flourish in the ecosystem. Hemp contains about 100 different types of terpenes [27,31]. These terpenes give it many fragrant qualities and can affect the medicinal qualities of the CBD-type cannabis [27,31]. They can also interact with the neurotransmitter receptors and increase the permeability of the blood–brain barrier. Thus, terpenes have been demonstrated to function in concert, enhancing the therapeutic potential of the whole-hemp products [21]. Monoterpenes and sesquiterpenes are the two primary terpene subtypes found in hemp [31]. Monoterpenes are compounds with ten carbons, including β-myrcene, pinene, terpinolene, ocimene, and limonene. β-caryophyllene is the most prevalent sesquiterpene [31]. The sedative effects of hemp are believed to be attributed to β-myrcene. Pellati et al. [17] reported that β-myrcene also possesses anti-inflammatory and anxiolytic characteristics. There is anti-proliferative activity in β-caryophyllene [17,31]. According to a study by Głodowska and Łyszcz, [21], terpinolene has antibacterial properties. It was found to inhibit the growth of Gram-positive and Gram-negative bacteria as well as the fungus yeast.

## 5. Biological Activity of *Cannabis sativa* Compounds

Studies with *C. sativa* have shown several biological activities, such as antibacterial, anticoagulant, antidiabetic, anticancer, anti-inflammatory, analgesic, and neuroprotective activities, as well as activities against neuropathic pain. 

### 5.1. Antimicrobial Activity

It has been documented that essential oils and organic extracts of various *C. sativa* components exhibit antibacterial action against a variety of microbes. Nevertheless, the level of antibacterial activity differs between cultivars [32], depending on the portion of the plant utilized, the manner of extraction, and the kind of extract. The hydroalcoholic extract from the seed was tested against a variety of Gram-positive and -negative bacteria, including *Bifidobacterium breve*, *B. longum*, *Salmonella typhimurium*, *Enterobacter aerogenes*, *Enterococcus faecalis*, *Limosilactobacillus reuteri*, *Lacticaseibacillus paracasei*, and *Levilactobacillus brevis*. The results obtained indicated a low level of antibacterial activity, with minimum inhibitory concentrations (MICs) greater than 1 mg/mL [33]. Anjum’s study examined the antibacterial properties of cannabis leaves by testing four extracts prepared with acetone, chloroform, ethanol, and water against three different bacterial strains: *Pseudomonas aeruginosa*, *Staphylococcus aureus*, and *Escherichia coli* [34]. The four extracts displayed commonalities in the results, with an up to 19 mm zone of inhibition (ZI). Furthermore, the findings reported by Manosroi et al. [35] demonstrated the effectiveness of ethanolic extract against *Streptococcus mutans* as an antibacterial agent, with a ZI value of 1.33 ± 0.58 mm. Furthermore, the antibacterial activity of the essential oil from the aerial portion gave MIC values of 64 and 38 mg/mL, respectively, against *Helicobacter pylori* and *Klebsiella pneumonia*, respectively, while the volatile terpenes of cannabis exhibited varying degrees of activity in relation to the targeted bacterial strains. Additionally, a more moderate inhibitory activity was observed against *E. coli*, *P. aeruginosa*, and *Bacillus subtilis*, with a MIC of 1.2 mg/mL [36]. In another study, antibacterial activity was observed against *Micrococcus luteus* and *S. aureus*, with a MIC of 4.7 mg/mL for both strains. Numerous biological studies have demonstrated that volatile terpenes of cannabis are potent antibacterial agents [37,38,39].

### 5.2. Anticoagulant Activity

It has been proposed that anticoagulant plants serve as herbal treatments that may help to find novel therapeutic agents to treat disorders associated with thrombosis. To ascertain the potential anti-prothrombotic impact of cannabis leaf metabolites, blood coagulation tests were carried out, focusing on the three primary cannabinoids: THC, CBD, and CBN [40]. It was revealed that two cannabinoids, THC and CBN, displayed considerable IC_50_ values of 87 and 83 μg/mL. THC exhibited the maximum activity, measuring 1.79 mg/mL, while CBN showed lower activity, as indicated by a high IC_50_ value of 9.89 mg/mL. To ascertain the clotting times, this study also included an in vivo test conducted on overweight rats. Consequently, the rats given cannabis showed a 50% efficiency rate with clotting that was twice as high as the control groups, indicating that cannabinoids may have a useful anticoagulant effect.

### 5.3. Antidiabetic Activity

Diabetes is a complicated metabolic disease that progresses over time and is defined by unusually high blood glucose levels. Another name for this illness is hyperglycemia [41]. Ninety percent of those affected globally have type 2 diabetes, which is defined as non-insulin-dependent diabetes [41]. The World Health Organization (WHO) states that diabetes is a chronic illness brought on by insufficient insulin production by the pancreas or improper insulin utilization by the body. The hormone insulin is responsible for controlling blood sugar levels. The standard method for assessing antidiabetic activity is to quantify the suppression of amylase [42]. Typically produced by the pancreas, this enzyme is essential in raising blood sugar levels because it breaks down dietary carbohydrates like starch into simple monosaccharides in the digestive tract. This is followed by additional α-glucosidase degradation into glucose, which is absorbed and enters the bloodstream. Thus, blocking the actions of the enzymes α-amylase and α-glucosidase might impede the breakdown of carbohydrates, postpone the absorption of glucose, and ultimately lower blood glucose levels [43]. With a value of 3.77 mmol ACAE/g oil, the study by Zengin et al. [44] demonstrated that the essential oils from the cannabis aerial portion demonstrated antidiabetic activities against the α-glucosidase enzyme. Additionally, this essential oil was tested against α-amylase; however, no noteworthy results were seen.

### 5.4. Anticancer Activity

Globally, cancer continues to be a leading cause of morbidity and mortality. Currently, traditional methods including radiotherapy, chemotherapy, and surgery are used to treat cancer, leaving affected individuals with adverse health impacts [45]. In view of these challenges, there have been deliberate attempts to explore cannabis and other natural resources for less toxic and more potent anticancer agents. In vitro and in vivo experiments on a variety of cancer cell lines, including breast [46], prostate [47], cervix [48], brain [49], colon [50], and leukemia/lymphoma [51], have shown the unambiguous efficacy of various cannabis-derived chemicals. Numerous in vivo and in vitro investigations have shown how phytocannabinoids affect the growth of tumors. According to these findings, at concentrations ranging from 5 to 65 µm, some cannabinoids, like Δ^9^-THC and CBD, cause apoptosis and limit proliferation in a variety of cancer cell lines [46,52,53,54,55,56,57]. Additionally, combining specific phytocannabinoids enhanced the anticancer potential of cannabis products. For instance, research by Armstrong et al. [52] showed that melanoma cell mortality was higher when CBD and Δ^9^-THC were combined than when Δ^9^-THC was used alone. Phytochemicals found in the cannabis plant, particularly cannabinoids, have little differential activity and are generally non-selective in their effects on cancer cells compared to normal cells. As a result, scientists are interested in synthesizing lead compounds based on a molecule’s natural backbone and extracting bioactive phytochemicals from cannabis that have strong anticancer effects.

### 5.5. Anti-Inflammatory and Analgesic Activities

Numerous constituents of cannabis have demonstrated potent anti-inflammatory properties. *Cannabis* seeds have been reported to reduce inflammation, particularly when applied to primary human monocytes treated with lipopolysaccharide (LPS). The seeds lowered TNF-α gene expression and IL-6 gene secretion. Moreover, cannabinoids demonstrated considerable anti-inflammatory action by inhibiting the synthesis of pro-inflammatory cytokines and chemokines and may be useful in treating inflammation-related illnesses [58,59]. Any procedure whose main goal is to lessen pain is referred to as an analgesic action. This can refer to not only medication but also any other technique intended to produce analgesia, or the elimination of pain perception [60]. Since opioids are the only treatment available for severe pain, cannabis has been identified as a much-needed substitute for opioids in terms of its analgesic properties [61]. Cannabinoids from cannabis function as opioid-sparing drugs, combining the benefits of opioids with the ability to reduce dosages and side effects associated with long-term opioid therapy [62]. Therefore, it is worthwhile to give careful thought to the prudent use of cannabis-based medications to relieve patients who are experiencing excruciating pain.

### 5.6. Neuroprotective Activity

The terpenes and cannabinoids of cannabis have also been shown to have neuroprotective qualities. In Pheochromocytoma-12 cells (PC-12), the neuroprotective properties of 17 substances found in *C. sativa* aerial parts were assessed. These compounds included ferulic acid, (*E*)-methyl-p-hydroxycinnamate, and p-hydroxybenzaldehyde, which also showed additional protective benefits against H_2_O_2_-induced damage [63]. Furthermore, Landucci et al. [64] demonstrated that suitable concentrations of CBD or CBD/THC ratios can represent a viable therapeutic intervention in the treatment of post-ischemic neuronal death. Di Giacomo et al. [65] reported the neuroprotective and neuromodulating effects induced by CBD and CBG in rat Hypo-E22 cells and isolated hypothalamus. A different study by Esposito et al. [66] emphasized the significance of CBD as a potentially useful novel medication that can lessen amyloid-induced neuroinflammatory reactions. Additionally, utilizing immuno-histochemistry analysis, the work by Perez et al. [67] detailed the neuronal counts of both motor and sensory neurons following CBD treatment. The results obtained indicated a 30% improvement in synaptic preservation on the spinal cord for the group receiving CBD treatment, indicating a moderate neuroprotective impact.

### 5.7. Neuropathic Chronic Pain Management

Positive (somatosensory function gain) and negative (somatosensory function loss) sensory sensations and indicators are commonly associated with neuropathic pain (NP) [68]. Either peripheral or central NP disorders can lead to chronic NP. The most recent classification of peripheral neuropathy NP was released by the International Association for the Study of Pain (IASP); the subtypes of chronic peripheral NP that fall under this category are trigeminal neuralgia (TN), chronic NP following peripheral nerve injury, painful polyneuropathy, post-herpetic neuralgia, and painful radiculopathy [69]. There are three subcategories of chronic central NP: chronic central NP linked to multiple sclerosis (MS), chronic central NP related to spinal cord injury (SCI) or brain injury, and chronic central post-stroke pain [69]. Twenty patients with chronic fibromyalgia pain were included in experimental randomized placebo-controlled four-way crossover research conducted in the Netherlands to examine the analgesic benefits of inhaled therapeutic cannabis. In the trial, three cannabis strains totaling 100 mg were administered in a single day: Bedrocan (22.4 mg THC, 1 mg CBD), Bediol (13.4 mg THC, 17.8 mg CBD), and Bedrolite (18.4 mg CBD, 1 mg THC). A placebo was also administered. The tolerance to pressure pain threshold was significantly increased by Bedrocan and Bediol. The cannabis strain with high levels of THC and CBD (Bediol) showed the most significant impact. The analgesic effects of CBD in combination with a very low amount of THC (Bedrolite, which primarily comprises CBD) were not better than those of a placebo. This outcome is different from trials where individuals with chronic pain reported positive effects from CBD treatment; these effects were likely connected to better mood, anxiety, and sleep patterns [70].

A prospective non-randomized single-arm clinical trial that took place in Italy in 2018 examined data from 338 patients who suffered from radiculopathy, headaches, fibromyalgia, and different types of neuropathic pain [71]. For a period of 12 months, they were given a daily dosage of 5–40 mg of THC (many of the individuals needed 10 mg), which is equivalent to 28–210 mg of cannabis floss with 19% THC and 1% CBD. At follow-up appointments, the participants’ level of discomfort was assessed. Of the patients, 77 discontinued the study because there was minimal benefit, and 33 quit because of adverse events (AEs), which may have been brought on by the high proportion of THC in Bedrocan (19%). According to this study, cannabis treatment is beneficial in lowering pain intensity using a visual analog scale (VAS), chronic pain-related disability, and the anxiety and depression that follow, as measured by the Hospital Anxiety and Depression Scale (HADS), all without producing severe adverse events [71].

Table 1 summarizes the biological activities of different cannabinoids found in *Cannabis sativa*.

## 6. Global Scenario and Legality of *Cannabis*

The hemp industry, which promotes the growth, processing, and use of hemp and its products, has been encouraged and sustained in recent years by creative applications of hemp materials and the growing concern for the sustainable development of the agricultural bioeconomy, especially in Canada and Europe [86]. In the 1990s, hemp farming was legalized in both the EU and Canada. By 2008, the output of hemp in Canada had risen rapidly, following some initial market adjustments. Currently, Canada produces more than 20,000 hectares of hemp each year under license, and they are almost all for seed [86]. Europe has seen a rapid expansion in hemp farming, with acreage rising from 8000 ha in 2011 to over 33,000 ha in 2016 [87]. The primary growing regions in Europe include Romania, France, the Netherlands, and the Baltic States. Nonetheless, many other European nations have begun or increased their hemp farming, mostly to produce hemp used for both fiber and seeds. The shift in the rapidly growing hemp seed market and the decreasing quality fiber requirements for novel biomaterials are driving dual-purpose hemp production in Europe [88]. Cannabis is becoming more and more popular, with the global cannabis market having a net worth of USD 14.5 billion in 2018. Now that more than 50 nations have legalized cannabis in some capacity for medical purposes and 6 more have legalized cannabis for recreational use, this market is predicted to reach USD 107.67 billion by 2025 [89]. Each country has different laws governing the possession, distribution, and cultivation of cannabis, as well as the conditions under which it can be used medicinally and how it should be taken.

With cannabis legalization in these countries and more countries to pass bills legalizing its use, issues relating to health risk, abuse, and other psychosocial problems abound. Although overdoses of cannabis do not easily result in the respiratory depression that is often associated with opioid use, a significant number of deaths from cardiovascular diseases and hyperemesis syndrome have been reported to accompany the sustained, heavy use of cannabis [90]. Furthermore, the non-medical use of cannabis has been reported to increase the risk of motor vehicle injuries when used at 1–3 h before driving, while it also increases the propensity to produce babies with low birth weights when used habitually during pregnancy [90,91]. Additionally, adolescents who use cannabis are more likely than adults to develop addiction problems [92]. Coffey and Patton have reported that habitual hemp use may contribute to cognitive impairment [93], early dropping out of school [94], the use of other illicit drugs, the development of schizophrenia and affective disorders [95], and suicidal thoughts [96]. Other problems associated with cannabis dependence include anxiety, depression, psychotic symptoms, and adverse gastrointestinal symptoms [97,98].

**Figure 3:**
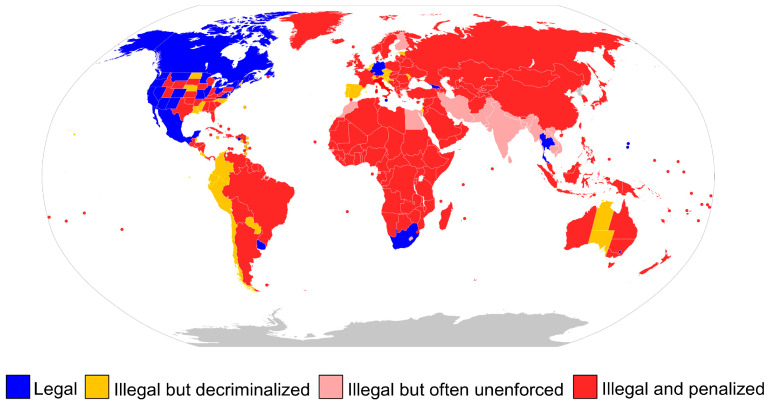
The legality of consuming cannabis globally for recreational purposes [104].

Three United Nations treaties—the 1968 Convention Against Illicit Traffic in Narcotic Drugs and Psychotropic Substances, the 1971 Convention on Psychotropic Substances, and the 1961 Single Convention on Narcotic Drugs—regulate these practices in many countries [99,100]. Under the Single Convention, cannabis was reclassified in 2020 from a Schedule I and IV drug to a Schedule I only substance (with Schedules IV, I, II, and III being the tightest-controlled to least) [101,102]. Although cannabis is classified as a Schedule I substance under the treaty, some countries may permit its medical use, even though it is highly addictive and has a high potential for abuse [102]. Although a few countries have decriminalized the use of cannabis by making simple possession of it an infraction that is treated like a minor traffic offense, many nations forbid its recreational use. Some countries in the Middle East and the Far East have much harsher laws, such as those that punish even minor amounts of possession with years in prison [103]. Canada, Georgia, Germany, Luxembourg, Malta, Mexico, South Africa, Thailand, and Uruguay, together with 24 states, 3 territories, and the District of Columbia in the United States, and the Australian Capital Territory in Australia, are among the nations that have legalized cannabis for recreational purposes (Figure 3).

**Figure 4:**
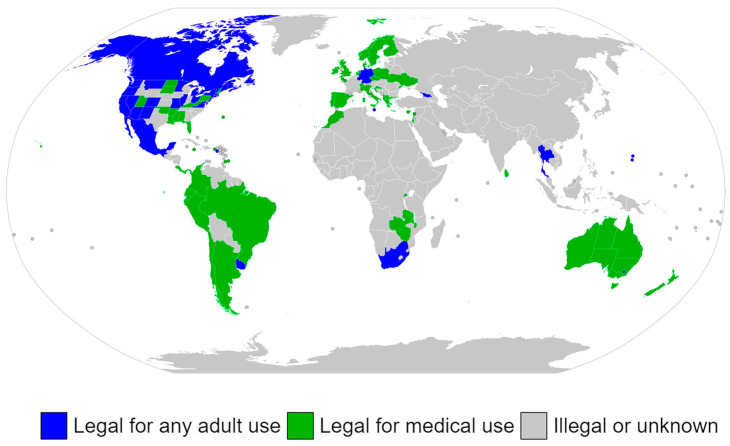
The legalization of cannabis globally for therapeutic purposes [106].

Three countries—Canada, Thailand, and Uruguay—as well as all subnational U.S. jurisdictions that have legalized possession of cannabis apart from Virginia and Washington, D.C., have permitted the commercial selling of cannabis for recreational use. Many nations have also embraced a policy of lax policing, most notably the Netherlands, where the sale of cannabis is allowed in establishments with a license [105]. Albania, Argentina, Australia, Barbados, Brazil, Canada, Chile, Colombia, Costa Rica, Croatia, Cyprus, Czech Republic, Denmark, Ecuador, Finland, Georgia, Germany, Greece, Ireland, Israel, Italy, Jamaica, Lebanon, Luxembourg, Malawi, Malta, Mexico, the Netherlands, New Zealand, North Macedonia, Norway, Panama, Peru, Poland, Portugal, Rwanda, Saint Vincent and the Grenadines, San Marino, South Africa, Spain, Sri Lanka, Switzerland, Thailand, Ukraine, the United Kingdom, Uruguay, Vanuatu, Zambia, and Zimbabwe are among the nations that have legalized cannabis for medical purposes (Figure 4). Others have more stringent regulations that only permit the use of specific medications made from cannabis, like Epidiolex, Marinol, and Sativex [106]. While the use of cannabis for medical purposes is now permitted in 38 states, 4 territories, and the District of Columbia in the U.S., it is still illegal at the federal level [106,107].

## 7. Cannabis as a Food or Beverage

### 7.1. Cannabis or Hemp Food

For decades, hemp has been a valuable food source for humans. Hemp leaves, sprouts, and flowers can be eaten fresh in salads and juices [108]. The most common part of the hemp plant to be eaten is the seed. Three to six weeks after the female flower is fertilized, the real seed, an achene, matures. It is protected by a hard, inconspicuous pericarp [108]. With some notable variations among different genotypes, hemp seeds include roughly one-fourth proteins, one-fourth carbohydrates, and one-third fats. They also give 500–600 Kcal/100 g [15]. The fats found in hemp seeds are polyunsaturated. Seven different hemp seed cultivars were studied, and the results showed that “Finola” had the highest concentration of α- and γ-linolenic acids and the lowest concentration of oleic acid and saturated fatty acids like palmitic and stearic acids. The cultivars were “Bialobrzeskie”, “Felina 32”, “Tygra 75”, “Futura 27”, “Santhica”, “Fedora 17”, and “Finola” [15]. Hemp seed proteins are rich in arginine, an important amino acid with advantageous cardiovascular effects, and a good source of cystine and methionine, two amino acids that contain sulfur. Growing data suggest that hydrolyzed hemp seed proteins have antihypertensive properties, which may be mediated by the suppression of renin and the angiotensin-converting enzyme [109]. This justification led to the development of a human experiment investigating hemp protein powder as a dietary intervention for hypertension [109]. Additionally, hemp seeds can be used to create oil and flour with beneficial nutritional qualities. When hemp flour was combined with starch to generate gluten-free bread, the flavor and color palatability improved, along with the nutritional value, which included higher amounts of proteins and fiber [109]. The nutritious value of hemp flour crackers has also increased [110]. The most popular edible hemp derivative is hemp seed oil, which has potential applications in the cosmetics sector as a sun lotion because of its high vitamin E concentration (100 mg/100 mL) and ability to absorb UV radiation [111]. Hemp seed oil has a nice, nutty flavor with a slightly bitter aftertaste. It can be used in cooking instead of olive oil, which has been shown to have cardiovascular health benefits [112].

### 7.2. Hemp Beer

**Figure 5:**
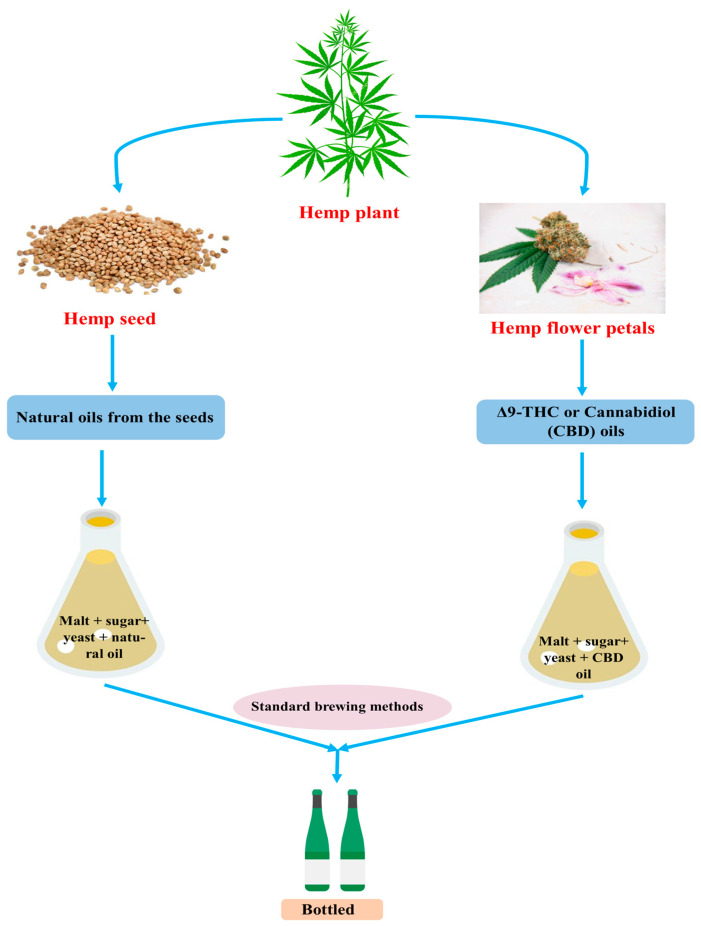
Hemp beer and cannabis-infused beer preparation.

In early 2017, hemp or cannabis-infused alcohol became popular. The federal ban on marijuana and the relaxation of regulatory barriers for cannabis plant parts other than the seed, which contains psychoactive Δ^9^-THC, led to this outcome [10]. Conventional brewing methods are used, together with the addition of hemp or cannabis. Whole and hulled hemp seeds, hemp seed oil, and hemp seed flour are foods derived from the hemp plant and are used as flavorings in beer [113]. There are no indications that the trend of cannabis-infused beer businesses blending their products will soon come to an end. Indeed, the availability of hemp beer and cannabis-infused beer has contributed to the rise in the popularity of craft beer. Hemp beer and cannabis-infused beer, also known as CBD- or Δ^9^-THC-infused beer, are two types of cannabis beer made from marijuana or hemp. Hemp beer uses natural oils from seeds to enhance its flavor, while CBD-infused beers use the standard brewing method with CBD oil (Figure 5). However, beers infused with THC are not alcoholic, as it is against the law to combine alcohol and THC, limiting their alcohol content to 0.5% by volume [10]. Due to their oily and hydrophobic nature, α-acids and cannabis are both poorly soluble in water, making it challenging to directly incorporate them into drinks. On the other hand, the α-acids undergo isomerization through boiling in a mildly acidic solution, resulting in iso-α-acids that possess an organoleptic bitter taste and are more soluble. Encapsulation, microemulsions, biopolymer nanoparticles, and filled hydrogel beads are the components of the colloidal delivery method for cannabis oil. These mechanisms guarantee cannabis distribution to the user by stabilizing the compounds against oxidation in a water-based beverage [114]. There are beers with Δ^9^-THC infusions available that have an alcohol-by-volume (ABV) of less than 0.5% and an Δ^9^-THC concentration ranging from 5 to 10 mg [115]. In contrast to Δ^9^-THC beers, CBD-infused beers include 10 milligrams of CBD and range in alcohol content from 3.5 to 6% ABV. When consumers consume CBD beers, they report “elevating” and “naturally calming” feelings, but THC beer causes intoxication, euphoria, lethargy, and occasionally cravings. The daily consumption of foods containing hemp should not amount to more than 12 μg of Δ^9^-THC per kilogram of body weight, as advised by the German Federal Institute for Health Protection of Consumers and Veterinary Medicine. This estimate indicates that 5 μg/kg of THC is present in beverages, both alcoholic and non-alcoholic [116].

## 8. Economic Importance of Cannabis

*Cannabis sativa* remains one of humanity’s useful natural resources because of its multipurpose applications in medicine, food, agriculture, and biotechnology. In the early 1990s, the Emerald Triangle region of Northern California was so popular for producing medical cannabis that the plant became the major driver of their economy, dominating other business activities, such as livestock and dairy production and lumbering [117]. By 1996, California had enacted the Compassionate Use Act, which was the first to permit legal access to and use of botanical cannabis for medicinal purposes under physician supervision [118]. With this, the state of California’s revenue generation through cannabis was USD 4 billion from USD 5.7 billion in sales in 2021 and has improved substantially to USD 5.1 billion in taxable sales by the end of 2023 [119]. The United States collected nearly USD 3 billion in marijuana revenue in 2022, while nationwide legalization could generate USD 8.5 billion annually for all states [119]. Similarly, Canada has generated about CAD 11 billion in sales since cannabis was legalized for recreational use in the country in 2018, with the industry contributing CAD 43.5 billion to the country’s GDP [120]. Australia remains in the early phase of development due to the export of cannabis only being legalized in 2018. The market potential in Australia is extremely high, with at least 200,000 patients eligible for medical cannabis, so the legal cannabis market is expected to increase from the fifty-two million Australian Dollars (AUD 52 m) that was achieved in 2018 to AUD 1.2 bn by 2025, with the potential to create over 10,000 new jobs across the horticultural, pharmaceutical, and medical industries [121].

Several partnerships and collaborations exist between Australian and Canadian companies. However, Canada struggles to meet the demand in a rapidly expanding recreational cannabis market and is thus seeking new suppliers [121]. Many Canadian entrepreneurs and investors have been streaming into Lesotho and a few other African countries where cannabis strains are present in abundance. Lesotho is a lower middle-income country, with a population of 2 million people, a per capita gross domestic product (GDP) of USD 999.7 as of 2022, and just USD 750 million in its internal reserve as of January 2024, which is also close to the country’s annual revenue base [122]. Lesotho has been largely growing cannabis since approximately the 1550s through illegal means and unlawfully using it for both medicinal and recreational purposes. It was only in 2017 that Lesotho started licensing cannabis companies and regulating the cultivation of cannabis for medical purposes [123]. So far, the cannabis economy in Lesotho is yet to be fully exploited due to cultural beliefs, stigmatization, and policy limitations [123]. The neighboring South Africa has a framework that legalizes the medical and recreational use of cannabis [124]. The country has put in place policies and recent regulatory changes which, for instance, limit the THC level in cannabis to 0.2% to prevent the misuse of cannabis and circumvent regulations around cultivation [125]. With the South African government’s plan to industrialize the sector by enacting a “Cannabis for Private Purposes Bill”, among other policies still in the pipeline, the hemp sector is projected to create over 130,000 new jobs and bring in more revenue to the country [125].

Therefore, more effort needs to be put in place for phase decriminalization, proper monitoring, and the regulation of cannabis among the top-producing countries. Through this, there would be numerous economic implications for states and their constituents, including raising tax revenues; generating economic activity such as new businesses, jobs, and income; and potentially altering migration patterns and demands for real estate. Overall, the continued growth and expansion of the cannabis market are almost certain because of the increasing demand for health-promoting, eco-friendly, and sustainable hemp products. These verifiable facts provide a formidable template for manufacturers, governments, and investors to tap into this lucrative and rapidly evolving market segment to help achieve economic stability for national development.

## 9. Conclusions and Future Prospects

It has been highlighted through this study that *Cannabis sativa*, though with restricted use in many countries because of its abusive and/or illicit use, can be a useful herbal ingredient due to its unique chemical diversity and notable biological properties. It has also been shown that the beneficial and/or detrimental effects of cannabis are dependent on the quality and quantity of its secondary metabolites, notably its cannabinoid contents (CBDs, THCs, CBGs, CBCs, and CBNs). With more countries legalizing cannabis cultivation and use, the public is becoming more aware of the negative psycho-social impact of cannabis and its usefulness in medicine, agriculture, food, and industrialization. Therefore, to fully harness the bioresources offered through cannabis for food, health and wellbeing, industrialization, and economic growth, there is a need for the routine scientific standardization of cannabis cultivars, with adequate monitoring, evaluation, regulation, and support.

In the future, it is our expectation that the hemp industry will not be limited to health or recreational use but will find applications as a valuable natural resource for cosmetics, textiles, and energy productions. There is, therefore, a need for many more countries to legalize cannabis and create a regulatory framework for the inclusion of CBD- and Δ^9^-THC-based cannabis. This will pave the way for the development of a wide range of cannabis- and/or hemp-infused products. Interestingly, this trend has already begun, such that CBD-, Δ^9^-THC-, and hemp oil products now feature prominently in the catalog of pharmaceutical products, contributing significantly to health and wellbeing as well as economic growth.

## Figures and Tables

**Table 1:** Therapeutic applications of cannabinoids found in *C. sativa*.

Compounds	Biological Application	References
Δ^9^-Tetrahydro-cannabinol (Δ^9^-THC)	Anti-inflammation, antianalgesic, antioxidant, anti-ulcers, antipruritic	[72,73,74]
Δ^9^-THCA	Anticancer and analgesic—stimulates CB1-dependent signaling in the N18TG2 neuroblastoma cell line	[75]
Cannabidiol (CBD)	Antianxiety, antinausea, antiarthritic, antipsychotic, anti-inflammatory, and immunomodulatory properties, antidiabetes, atherosclerosis, treatment of alzheimer disease, hypertension, metabolic syndrome, ischemia–reperfusion injury, depression, and neuropathic pain.	[76,77,78]
CBDA	Rescued memory deficits and reduced amyloid-beta and Tau pathology in an Alzheimer’s disease-like mouse model	[79]
Cannabinol (CBN)	Sedative, antibiotic, anticonvulsant, anti-inflammatory	[80]
CBNA	Antioxidant property due to free radical scavenging activity	[81]
Cannabichromene (CBC)	Anti-inflammatory/analgesic, antidepressant	[82,83]
CBCA	Rapid antibacterial activity against methicillin-resistant *Staphylococcus aureus*	[84]
Cannabigerol (CBG)	Antioxidant, anti-inflammatory, analgesic effect	[74,82]
CBGA	Exhibits antioxidant activity manifested in its ability to scavenge free radicals, to prevent the oxidation process, and to reduce metal ions	[85]

## Data Availability

Not applicable.
